# Associations and Synergistic Effects for Psychological Distress and Chronic Back Pain on the Utilization of Different Levels of Ambulatory Health Care. A Cross-Sectional Study from Austria

**DOI:** 10.1371/journal.pone.0134136

**Published:** 2015-07-28

**Authors:** Kathryn Hoffmann, Wim Peersman, Aaron George, Thomas Ernst Dorner

**Affiliations:** 1 Department of General Practice, Center for Public Health, Medical University of Vienna, Vienna, Austria; 2 Department of Family Medicine and Primary Care, Ghent University, Ghent, Belgium; 3 Department of Community and Family Medicine, Duke Medical Center, Durham, NC, United States of America; 4 Institute for Social Medicine, Center for Public Health, Medical University of Vienna, Vienna, Austria; Tilburg University, NETHERLANDS

## Abstract

The aim of this analysis was to assess the impact of chronic back pain and psychological distress on the utilization of primary and secondary levels of care in the ambulatory health care sector in Austria - a country without a gatekeeping system. Additionally, we aimed to determine if the joint effect of chronic back pain and psychological distress was higher than the impact of the sum of the two single conditions. The database used for this analysis was the Austrian Health Interview Survey, with data from 15,474 individuals. Statistical methods used were descriptive tests, regression models and the calculation of synergistic effects. Both chronic back pain and psychological distress had a positive association with the utilization of the primary (OR for chronic back pain 1.53 and psychological distress 1.33) and secondary (OR for chronic back pain 1.32 and psychological distress 1.24) levels of the health care sector. In the fully adjusted model, the synergistic effect of chronic back pain and psychological distress was significant for the secondary level of care (S 1.99, PAF 0.20), but not for the primary level of care (S 1.16, PAF 0.07). Synergistic effects and associations for chronic back pain and psychological distress on the utilization of both the primary and secondary levels of the ambulatory health care sector were observed, particularly for the secondary level of care. Our results demonstrate the utilization of health care services settings by individuals with these conditions, and offer opportunities to consider reorganization and structuring of the Austrian health care system.

## Introduction

Chronic back pain (CBP) is a common condition as evidenced by literature estimating that approximately 20% of Europeans report chronic pain, with chronic back pain responsible for the majority of cases [1]. CBP decreases not only the quality of life for the individual, but is also a relevant societal challenge for the health care and economic system [1, 2]. Back pain is the major cause for loss in productivity [3] and sick leave [4], and is a major cause for disability pension [4–6] and presenteeism [3, 7]. In addition, mental health problems have been shown to impact the extent of sick leave taken by individuals [8]. Literature recognizes that depression is strongly associated with chronic pain [9], especially with increased prevalence of back pain. However, evidence is still conflicting with regard to the timing and causality of this relationship [10]. Moreover, both depression and other nonspecific and sub-acute psychological conditions such as psychological distress (PD) are associated with an increased prevalence of musculoskeletal problems. Dorner and colleagues found, for example, that social stress in the workplace more than doubled the probability for musculoskeletal problems in males, and sexual dissatisfaction was correlated with a three-fold increase in reporting of musculoskeletal pain [11]. PD, as it is defined in this publication, is a sub-acute condition which results from a persons´ non-ability to cope with personal, social, or occupational demands [8, 12].

It is well known that individuals with chronic pain make more contact points with the health care system [13, 14]. In contrast, literature suggests that persons with psychosocial conditions, such as partnership problems and social stress are less likely to visit the primary health care system for those psychosocial reasons, but more for physical complaints [15–17]. Primary health care is recognized as a focal point for customized and effective management of psychosocial conditions, especially those in combination with chronic pain [18–23]. In the United States, a country without a gatekeeping system like Austria, those with PD that have no primary care physician, or those who cannot afford one, are more likely to use emergency services for non-emergency reasons [24].

There is limited evidence for an additive effect of depression and pain on the utilization of specialist care. For example, one study in patients with spinal injuries in the United States showed that the additive effect of pain and depression were associated with a higher utilization of specialist care than the sum of both effects alone [25]. Similar observations were found in rural settings in Canada, where the presence of both chronic pain and depression led to a higher primary health care utilization [26]. In addition, a larger Swedish cohort study recently showed a synergistic effect of back pain and common mental disorders that lead towards disability pension [27]. However, little is known about CBP and PD on the utilization of the different levels of the ambulatory health care system in a European country without a gatekeeping system, and even less about the possibilities of a synergistic effect.

In this study, the term primary care is used to reference ambulatory general practitioner (GP) offices. Meanwhile, the secondary level of care includes those that are composed of specialist practitioners such as ophthalmologist, dermatologist, orthopaedist, internal medicine specialist, ENT specialist, gynaecologist, or urologist. The reason we were interested in both levels of ambulatory care was that in Austria, with some exceptions, patients have free access and financial coverage to both the primary and secondary level of care. Patients make individual determinations as to which level of care she or he consults [28]. Therefore, Austria is an ideal setting to observe a possible synergistic effect of PD and CBP on the utilization of different levels of care in the ambulatory sector.

It was the aim of this analysis to assess the impact of CBP and PD on the utilization of the primary and secondary levels of care in the ambulatory health care sector. Additionally, the intent was to evaluate if the joint effect of CBP and PD is higher than the impact of the sum of the two single conditions.

## Materials and Methods

### Subjects

The data analyzed for this paper were part of a database of the Austrian Health Interview Survey (AT-HIS) 2006–07 [29], the most recent AT-HIS data available. The interviews for this survey were conducted face-to-face using the CAPI (computer assisted personal interviewing) method by a total of 137 specially trained interviewers. Those persons that agreed to participate were interviewed between March 2006 and March 2007 and had to be older than 15 years of age. The survey was commissioned by the Austrian Federal Ministry of Health, Family and Youth, was carried out by Statistics Austria and designed based on the European Core Health Interview Survey (EC-HIS) [30].

The interview questionnaire consisted of 450 items regarding diseases, health behavior, health care utilization, subjective health status and quality of life, as well as socio-demographic and socio-economic variables.

The response rate was 63.1%, which means that data of 15,747 subjects from the 25,130 interviewed were eligible for the analysis. The sample was stratified by geographic region, with equal numbers of subjects being included in each region.

This secondary analysis was designed in accordance with the STROBE criteria for cross-sectional studies [31, 32], the completed STROBE checklist has been submitted as S1 Table.

### Variables

#### Dependent variables

The variables of the utilization of the healthcare system were defined as dependent variables. The utilization of the primary level of care was assessed with the question “within the last 4 weeks, did you turn to a GP?” The utilization of the secondary level of care in the ambulatory sector was assessed with the two questions that were merged together to this one variable “within the last 4 weeks, did you consult any specialist in the ambulatory sector (ophthalmologist/ dermatologist/ orthopaedic specialist/ specialist in internal medicine/ ENT specialist/ gynaecologist/ urologist/other specialist)?” and “within the last 4 weeks, did you consult any outpatient clinic?” For all questions the answer categories were “yes”, “no”, and “don´t know”. The dichotomous variable GP or specialist consultation was built by taking all “yes” answers as positive consultations and the remainders as “no” answers.

#### Independent variables

Chronic back pain (CBP): The variable relating CBP was introduced with the sentence: “Now I will present you a list of chronic conditions and diseases. Please, do only consider long-term problems and not acute symptoms or diseases.” The question concerning the chronic condition was as follows: “Did you ever have chronic back pain (pain of the lumbar or thoracic vertebral column, or of the neck?” If the participant answered with “yes” the next question was “Did you experience this condition within the past 12 months?” Here, the answer options were “yes”, “no” and “don´t know”. The answer options were dichotomized into the “yes” answers, including all persons with CBP within the past 12 months, and all other answer options.

Psychological distress (PD): PD was surveyed with the questions assessing “general mental health, covering PD” of the standardized and validated Short-Form-36 Health Survey (SF-36) questionnaire [33]. The questions were “how often during the last four weeks did you feel “nervous”, “down”, “calm and peaceful”, “downhearted and blue” and “happy”. Possible answers were “always”, “mostly”, “some of the time”, “little of the time”, and “never”. As recommended, the items were scored according to the “Rand approach”, which recodes the answers of the questions into a 0–100 score with coding in steps of 25, ensuring that higher scores represent better mental health [34]. In a further step the scored persons were split into two groups. As there are no normative values about what makes a ‘good’ or a ‘bad’ emotional wellbeing and no cut-off scores can be found in literature. The first group consisted of people with a score below the median, meaning the presence of higher PD. Meanwhile, group two included those individuals with a score equal to or higher than the median, suggesting less emotional distress than the remainder. The median was calculated separately for men and women. Women were split into two groups in accordance with their median (PD < 80 score points) and men according to their median (PD < 85 score points) respectively. Furthermore, four groups were built out of the PD and CBP variables: the first group consisted of subjects with both CBP and PD, the second group of subjects with CBP and no PD, the third group of persons with PD and no CBP and the fourth group of persons without either CBP or PD.

Control variables: These included sex, age, educational level, country of origin, place of residence, presence of a partnership, Body Mass Index (BMI), and recent smoking status. Highest educational status was assessed in three categories: primary education (up to the age of 15), secondary education (apprenticeship or secondary school) and tertiary education (university or any further education). Migration status was surveyed with the question “What is your country of birth?” The variable was stratified in three categories: Austria, European Union (EU) 27 countries except Austria but including the European Free Trade Association countries (EU27+), and all other countries. It was not possible to build the variable EU 28 countries, which would be correct as of the 1^st^ of July 2013, because of pre-clustered data in the AT-HIS database. The place of residence was surveyed with the question “In which federal state do you live?” BMI was calculated from the weight and height of the subjects using the special BMI formula (kg/m²) and recent smoking status was assessed with the question “Do you smoke recently?” with the answer categories “yes, every day”, “yes, sometimes” and “”no”. The variable was dichotomized by taking all yes answers together to form one answer category.

In addition, the existence of other chronic diseases within the last 12 months was taken into account. Other chronic diseases surveyed were assessed with the following questions “Within the last 12 months did you have allergic asthma/ other forms of asthma/ allergies/ diabetes/ cataract/ tinnitus/ hypertension/ myocardial infarction/ insult or cerebral haemorrhage/ chronic bronchitis or emphysema/ aconuresis/ cancer/ gastric or intestinal ulcer/migraine?” with the answer categories “yes” or “no”. This enumeration represents all chronic diseases that were asked in this survey with the exception of chronic pain of the vertebral column.

### Statistical analysis

Descriptive analyses are presented as absolute and relative numbers. To find statistically significant differences between subgroups the Chi-Square test was applied. In addition, means and standard deviations were presented for the scale variables. Moreover, we applied logistic regression models. In a first unadjusted crude model, PD and CBP were analyzed as independent variables, one after the other for the utilization of GPs and specialists. In a second adjusted model, the socio-demographic variables (age, educational level, place of residence, country of origin, and partnership status) were added as control variables. In a third model, performed in addition to the variables of the second adjusted model, all chronic somatic diseases surveyed that were other than CBP and PD were included, as well as Body Mass Index (BMI) and smoking status. In a forth fully adjusted model, CBP was added as control variable for PD, and PD as control variable for CBP. The results of all logistic regression models are presented as odds ratios (OR) with 95% confidence intervals (95% CI). We included socio-demographic and health data as control variables for the logistic regression models, as it is well-known that these factors highly influence the utilization of the health care system [14, 35, 36]. Following, the calculation of the synergistic effect of PD and CBP on the utilization of the ambulatory health care sector levels in these models was performed.

Synergistic effects were identified with synergy index (S), population attributable fraction (PAF), and relative excess risk due to interaction (RERI). The interaction effect is calculated as the deviation from an additive model. The S is the ratio of the observed effect with combined exposure of both risk factors to the effect expected with independently acting risk factors. It is calculated with the formula S = (OR CBP & PD– 1) / [(OR CBP & no PB– 1) + (OR PB & no CBP– 1)]. PAF due to interaction shows the proportion of health care utilization attributable to the interaction effect only (without the effect of the both individual observed factors). PAF is computed with the formula PAF = [(OR CBP & PD)–(OR CBP & no PD)–(PR PD & no CBP) +1] / (OR CBP & PD). And RERI is a metric of departure from additivity of effects. It is computed with the formula RERI = (OR CBP & PD)–(OR CBP & no PD)–(PR PD & no CBP) +1. S would be equal 1, and PAF and RERI would be equal 0 for factors acting independently without interaction. S would be equal 0 and PAF and RERI would approach minus infinity, when two factors completely neutralize each other. If there is a synergistic effect, S is higher than 1 and PAF and RERI are higher than 0, and in the extreme, when two factors produce no effect independently but a measurable effect together, S and RERI would approaches infinity, and PAF would approach 1. Confidence intervals for S, PAF, and RERI are calculated using the delta method, a straight-forward Taylor expansion of the variances and covariances [37].

Moreover, in order to account for the stratification of the sample the data were weighted by sex, age and geographic region to increase representativeness.

Calculations were done using SPSS version 22.0. SI, RERI and PAF were calculated with an MS Excel document.

### Ethical considerations

Data acquisition was performed by Statistics Austria commissioned by the Austrian Federal Ministry of Health, Family and Youth. Participants provided their verbal informed consent before they participated in the survey. Participants between 15 and 18 years of age had to additionally give their verbal informed consent before they participated in the survey. The authors were not involved in the data acquisition process; rather they received the data of the survey after the collection was completed to perform secondary analyses. However, the data acquisition process as well as the information about the verbal informed consent was stated at the Ethics Committee of the Medical University of Vienna for receiving the approval for the secondary data analysis. The secondary analysis for this study was approved by the Ethics Committee of the Medical University of Vienna (EC # 770/2011).

## Results

### Sample

Data from 8,021 women (51.8%) and 7,453 men (48.2%) were analyzed. Of all women surveyed, 40.8% (n = 3,275) were considered as having PD and 34.5% (n = 2,766) responded as having CBP. In contrast, 44.4% (n = 3,311) of all males were considered as having PD and 30.5% (n = 2,269) experienced CBP.

Table 1 shows the distribution of the demographic and health variables for the whole sample.

**Table 1:** Characteristics of respondents (n = 15474), values are numbers (%) unless stated otherwise.

Variable	Sub-variable	n (%)
Gender	male	7453 (48.2)
	female	8021 (51.8)
Age (years)	15–34	4667 (30.2)
	35–54	5661 (36.6)
	55–74	3707 (24.0)
	75+	1439 (9.3)
Educational level	Primary	4188 (27.1)
	Secondary	9836 (63.6)
	Tertiary	1450 (9.4)
Country of origin	Austria	13025 (84.2)
	EU 27+	856 (5.5)
	Others	1593 (10.3)
Person with	no CBP & no PD	6628 (42.8)
	no CBP & PD	3811 (24.6)
	CBP & no PD	2259 (14.6)
	CBP & PD	2776 (17.9)
BMI (mean (SD))		25.2 (4.3)
GP visit		5607 (36.2)
Specialist visit		3800 (24.6)
Living in partnership		10186 (65.8)
Currently smoking		4031 (26.0)
Allergies		2423 (15.7)
Allergic asthma		424 (2.7)
Other forms of asthma		313 (2.0)
Diabetes		862 (5.6)
Cataract		616 (4.0)
Tinnitus		946 (6.1)
Hypertension		2927 (18.9)
Myocardial infarction		76 (0.5)
Insult or cerebral hemorrhage		125 (0.8)
Chronic bronchitis or emphysema		596 (3.9)
Aconuresis		814 (5.3)
Cancer		182 (1.2)
Gastric or intestinal ulcer		407 (2.6)
Migraine		2326 (15.0)

BMI: Body-Mass-Index; CBP: chronic back pain; GP: General Practitioner; PD: psychological distress; SD: Standard Deviation

### Impact of PD and CBP on GP and specialist visits

Table 2 shows the different models for the impact of PD and CBP on the utilization of the primary and secondary level of ambulatory care. Both PD and CBP showed a significant positive association with GP visits and specialist consultations, even after the full adjustment in model IV. This means that, after the full adjustment for other chronic diseases and socio-demographic variables, the probability of persons with both CBP and PD, respectively, consulting a specialist was increased significantly, with an OR of 1.68. Further, the probability of a person with only CBP consulting a specialist showed an OR of 1.19, compared to subjects without CBP and without PD (Table 3).

**Table 2:** The different models for the impact of PD and CBP on the utilization of GP and specialist visits.

Level of care		Crude model I	Model II^a^	Model III^b^	Model IV^c^
		OR (CI 95%)	OR (CI 95%)	OR (CI 95%)	OR (CI 95%)
**Primary**	PD	1.86 (1.74–1.99)	1.66 (1.54–1.78)	1.39 (1.29–1.50)	1.33 (1.24–1.44)
	CBP	2.38 (2.22–2.56)	1.86 (1.73–2.01)	1.58 (1.46–1.71)	1.53 (1.41–1.65)
**Secondary**	PD	1.43 (1.33–1.54)	1.43 (1.32–1.54)	1.28 (1.18–1.38)	1.24 (1.15–1.35)
	CBP	1.64 (1.52–1.77)	1.50 (1.38–1.62)	1.35 (1.24–1.47)	1.32 (1.21–1.43)

^a^Model II: adjusted for socio-demographics (sex, age, education, country of origin, place of residence, living with partner)

^b^Model III: similar to model II with additional adjustment for each chronic somatic disease other than chronic pain of the vertebral column and psychological diseases surveyed, BMI, and recent smoking status

^c^Model IV: similar to model III and PSD with additional adjustments for CBP, and CBP additionally adjusted for PSD

PD: psychological distress; CBP: chronic back pain

**Table 3:** Synergistic effects for the four different CBP/PD groups for GP visits.

CBP	PD	GP consultations	Model I Crude	Model II^a^	Model III^b^
		% (n)	OR (95% CI)	OR (95% CI)	OR (95% CI)
+	+	55.9 (1,551)	3.64 (3.32–4.00)	2.67 (2.42–2.95)	2.03 (1.83–2.25)
+	-	42.9 (969)	2.16 (1.96–2.39)	1.74 (1.57–1.93)	1.54 (1.39–1.72)
-	+	36.2 (1,379)	1.63 (1.50–1.78)	1.53 (1.40–1.67)	1.34 (1.22–1.47)
-	-	25.8 (1,709)	1.0	1.0	1.0
S			1.48 (1.26–1.73)	1.32 (1.08–1.61)	1.16 (0.90–1.51)
PAF			0.23 (0.15–0.32)	0.15 (0.05–0.25)	0.07 (-0.05–0.19)
RERI			0.85 (0.51–1.19)	0.40 (0.12–0.68)	0.14 (-0.10–0.39)

^a^Model II: adjusted for socio-demographics (sex, age, education, country of origin, place of residence, living with partner)

^b^Model III: similar to model II with additional adjustment for each chronic somatic disease other than chronic pain of the vertebral column and psychological diseases surveyed, BMI, and recent smoking status

CBP: chronic back pain; GP: General Practitioner; PAF: population attributable fraction; PD: psychological distress; RERI: relative excess risk due to interaction; S: synergy index

### Synergistic effects for PD and CBP on GP and specialist visits

Tables 3 and 4 show that the respondents that reported to have had contact with GPs or specialists were nearly twice as high in the simultaneous PD and CBP group than in the group without PD or CBP. Additionally, they describe two findings each: First that the likelihood of consulting a physician is significantly higher for individuals with both conditions compared to individuals without these conditions, for both the primary and the secondary levels of care. Secondly, statistically significant synergistic effects (S) were found for the group of subjects with PD and CBP related to the primary, as well as the secondary level of ambulatory care (Tables 3 and 4). However, for the primary level of care the synergistic effect was found to be significant until the full adjustment. The synergistic effect for PD and CBP for visiting a specialist was nearly twice as high as it would have been expected on the basis of the mere addition of the effects of the single PD and CBP variables. Further, the PAF shows that 20% of the specialist consultations can be explained by the synergistic effect.

**Table 4:** Synergistic effects for the four different CBP/PD groups for specialist visits.

CBP	PD	Specialist consultations	Model I Crude	Model II^a^	Model III^b^
		% (n)	OR (95% CI)	OR (95% CI)	OR (95% CI)
+	+	35.0 (972)	2.13 (1.93–2.35)	1.98 (1.78–2.19)	1.68 (1.50–1.87)
+	-	26.1 (589)	1.39 (1.24–1.55)	1.27 (1.14–1.43)	1.19 (1.06–1.34)
-	+	23.6 (899)	1.22 (1.11–1.34)	1.24 (1.13–1.37)	1.15 (1.04–1.27)
-	-	20.2 (1,340)	1.0	1.0	1.0
S			1.85 (1.33–2.58)	1.88 (1.29–2.74)	1.99 (1.15–3.44)
PAF			0.24 (0.14–0.34)	0.23 (0.13–0.34)	0.20 (0.09–0.32)
RERI			0.52 (0.29–0.75)	0.46 (0.24–0.68)	0.34 (0.13–0.54)

^a^Model II: adjusted for socio-demographics (sex, age, education, country of origin, place of residence, living with partner)

^b^Model III: similar to model II with additional adjustment for chronic somatic diseases others than chronic pain of the vertebral column and psychological diseases, sum of chronic diseases, BMI, and recent smoking status

CBP: chronic back pain; PAF: population attributable fraction; PD: psychological distress; RERI: relative excess risk due to interaction; S: synergy index

## Discussion

These findings represent the first analysis of associations and synergistic effects of PD and CBP on the utilization of both the primary and secondary level of ambulatory care in a country without a gatekeeping system.

The finding that both single factors had a positive association with the utilization of the health care sector would be expected. However, it is interesting that a nonspecific emotional factor, such as PD, significantly increased the likelihood of consulting the secondary level of care in a country without gatekeeping, even after the full adjustment with an OR of 1.24 (Table 2).

The synergistic effect revealed that if both factors of PD and CBP occur together, the need of patients for care at all levels was much higher than the mere addiction of the effect of the individual factors. This was nearly double the effect that the addition of the single factors displayed, with a synergistic index of 1.16 for the primary level of care and 1.99 for the secondary level of care (Tables 3 and 4). Particularly for the secondary level of care, this finding is remarkable; indicating that if CBP occurs together with an unspecific psychological condition, about 20% of those specialist consultations could be explained by the interaction effect of CBP and PD alone. In addition, both PD and CBP showed a significant positive association with GP visits and specialist consultations. This means that CBP and an unspecific condition such as PD increased the probability of presenting to a GP or specialist significantly (Table 2).

After adjustment for other chronic diseases, the significance of the synergistic effect vanished for the primary level of care. This suggests that a large proportion of primary health care utilization in these patients was probably mediated alongside other chronic diseases. CBP and PD patients very often had other chronic conditions, especially, those with both conditions simultaneously, as well as those patients with CBP only.

Our findings implicate consequences for transforming the primary level of care. The synergistic effect should be seen as a special window of opportunity, due to the fact that the likelihood to visit a GP with these conditions is very high (Tables 2 and 3). Recent findings suggest that PD as a non-specific psychological condition, and CBP as a chronic condition, could be best managed at the primary level of care. This is due to the fact that primary health care is centered around core competences of person-centered care, comprehensiveness, continuity, and coordination of care, which could provide an ideal framework for addressing these bio-psycho-social problems and better coordinate the complex care strategy needed [38–40]. Stein and colleagues found that the most important element for care of chronic pain patients in Austria is a comprehensive discussion with their physician; which was observed as more important than a reduction in pain intensity. More efforts have to be made to educate and inform chronic pain patients adequately from the doctor's side considering their psycho-social context [41]. One other important issue seems to be to ask and apply relevant diagnostics and evaluation for co-morbidities. To be able to make use of this window of opportunity the primary level of health care should be organized and supported to strongly enable the application of the core competencies of primary care. Unfortunately, this is not completely the case in the health care delivery system in Austria, especially with regard to the core competencies for coordination of care and comprehensiveness [28, 36, 40, 42].

Our results could implicate additional consequences for the secondary level of ambulatory care. It seems that in a country without a gatekeeping system, where the access to the secondary level of care is free, persons with CBP and/or PD have a higher probability to present to the secondary level of care (Tables 2 and 4). This raises several questions, including as to whether the secondary level of care is the best point of care for persons with complex psychological and chronic conditions as well as multi-morbidities [43, 44]? Further, what is the underlying reason for consultation of specialists? Is it an integrated care concept, or just the perception of the patients that believe that the secondary level of care is a more sophisticated level of care? Unfortunately, the study design and questionnaire does not allow such analyses. However, it could be speculated that at least some part of the consultations at specialists were due these patient perceptions, as Austria is a secondary care focused country, and the overall utilization of the second level of health care is quite high [36, 45]. In this case it could be of benefit for patients to be guided through the complex health care system with the support of a primary care provider, preferentially through structural incentives or a gatekeeping system.

The strengths of the present analysis were the analytical design and the large sample size, which allowed adjustment for possible confounders. Additionally, the large number and random selection of participants offers a high external validity of the results, and in particular for Austria. The utilization of a consistent survey-interview-team and a standardized questionnaire increased the probability for data consistency. One major limitation is the challenge of diagnosing PD: for measuring psychological distress we used the “general-mental-health”-questions of the Short-Form-36 Health Survey which covers psychological distress. However, this tool cannot distinguish from more severe or acute forms of distress or psychopathologies or from a mixture of somatic and psychological symptoms. It would have been of benefit to additionally use other tools like the Four-Dimensional-Symptom Questionnaire (4DSQ) to be able to better distinguish the more severe and acute forms of mental health disorders in distressed GP patients as recommended by Terluin et al. from the sub-acute psychologically distressed patients [46]. This challenge could have led to an overestimation of persons with sub-acute PD. Another important limitation which could have influenced the results is that different time frames were used throughout the given questionnaire design; with a 12 month cutoff assessed for the existence of CBP and a four week one for PD in conjunction with utilization of the two levels of the ambulatory health care system. This cutoff may have underestimated the number of patients seeking care for PD, or otherwise captured a larger number of patients that sought care for CBP, in comparison. On the other hand, PD has the same timeframe as the health care utilization question and CBP is by definition chronic which implies a longer period than 4 weeks to determine the presence meaning the influence of the results may be a limited one. Moreover, the analysis is cross-sectional and, therefore, no causal relationships can be drawn. Additionally, results are based on descriptive and self-reported survey data rather than administrative data [47]. Furthermore, the cut-off value for the two PD groups was based on the median, as no normative values for psychological distress could be identified in the literature. Finally, and very important, we had no knowledge about the reasons for and appropriateness of the consultations.

## Conclusion

Synergistic effects and associations for CBP and PD towards the utilization of both the primary and secondary level of the ambulatory health care sector were identified. In addition, persons with CBP and/or PD had more co-morbidities than persons without CBP and PD. Our results could yield consequences for the organization of the health care system. Particularly for primary level of care, these results could be seen as a window of opportunity, where the complex care strategy needed for psychosocial as well as chronic diseases and multi-morbidities could be emphasized.

This is particularly important, as patients have the freedom to access either level of care based on their individual preference. The results have profound implications on steering health system organization, where priorities can be made to improve avenues for patients to access care at the best point of entry into the system. Specifically, secondary levels of ambulatory care are often more costly, and may not lead to positive differences in outcomes. Counter, it has been observed that, for patients with concomitant PD and CBP, that the best point of care is most often a primary care setting.

To better direct patients towards the most appropriate health services, it would be valuable to avoid indiscriminate self-referral to secondary levels of care. To meet these goals, efforts must additionally be made to strengthen the quality of care at the primary level of care as well as align to meet recent recommendations. The findings of this study support recommendations for health care governance and health systems to improve coordination of person-centered care in Austria and similar countries.

## Supporting Information

S1 Table STROBE Statement—Checklist of items that should be included in reports of cross-sectional studies. (DOC)
